# Feasibility of a novel eHealth intervention for Parkinson’s disease targeting motor-cognitive function in the home

**DOI:** 10.1186/s12883-024-03614-2

**Published:** 2024-04-05

**Authors:** Jenny Sedhed, Hanna Johansson, Nina Andersson, Elisabet Åkesson, Elke Kalbe, Erika Franzén, Breiffni Leavy

**Affiliations:** 1 Stockholm Sjukhem Foundation, R&D unit, Stockholm, Sweden; 2 Department of Neurobiology, Care Sciences and Society, Division of Physiotherapy, Karolinska Institutet, Alfred Nobels Allé 23, Huddinge, 141 83 Sweden; 3 Theme Women’s Health and Allied Health Professionals, Medical unit Occupational Therapy & Physiotherapy, Karolinska University Hospital, Stockholm, Sweden; 4 Department of Neurobiology, Care sciences and Society, Division of Neurogeriatrics, Karolinska Institutet, Stockholm, Sweden; 5 Medical Psychology | Neuropsychology and Gender Studies and Center for Neuropsychological Diagnostics and Intervention (CeNDI), Faculty of Medicine and University Hospital Cologne, University of Cologne, Cologne, Germany

**Keywords:** Cognition, eHealth, Exercise, Motor-cognitive exercise, Parkinson’s disease

## Abstract

**Background:**

Parkinson’s disease (PD) drastically affects motor and cognitive function, but evidence shows that motor-cognitive training improves disease symptoms. Motor-cognitive training in the home is scarcely investigated and eHealth methods can provide continual support for PD self-management. Feasibility testing is however required.

**Objective:**

To assess the feasibility (i) Recruitment capability (ii) Acceptability and Suitability (iii) Demand and Safety of a home-based motor-cognitive eHealth exercise intervention in PD.

**Methods:**

The 10-week intervention was delivered using the ExorLive® application and exercises were individually adapted and systematically progressed and targeted functional strength, cardiovascular fitness, flexibility, and motor-cognitive function. People with mild-to moderate PD were assessed before and after the intervention regarding; gait performance in single and dual-task conditions; functional mobility; dual-task performance; balance performance; physical activity level; health related quality of life and perceived balance confidence and walking ability; global cognition and executive function. Feasibility outcomes were continuously measured using a home-exercise diary and contact with a physiotherapist. Changes from pre- and post-intervention are reported descriptively.

**Results:**

Fifteen participants (mean age 68.5 years) commenced and 14 completed the 10-week intervention. In relation to intervention *Acceptability*, 64% of the motor sessions and 52% of motor-cognitive sessions were rated as “enjoyable”. Concerning *Suitability*, the average level of exertion (Borg RPE scale) was light (11–12). Adherence was high, with 86% of all (420) sessions reported as completed. No falls or other adverse events occurred in conjunction with the intervention.

**Conclusions:**

This motor-cognitive eHealth home exercise intervention for PD was safe and feasible in terms of Recruitment capability, Acceptability, Safety and Demand. The intensity of physical challenge needs to be increased before testing in an efficacy trial.

**Trial registration:**

This trial is registered at Clinicaltrials.gov (NCT05027620).

**Supplementary Information:**

The online version contains supplementary material available at 10.1186/s12883-024-03614-2.

## Introduction

The gradual onset of motor impairment during early Parkinson’s disease (PD) stages reduces movement capacity and partly accounts for why most people with PD (PwPD) fail to meet general physical activity recommendations for the maintenance of health [1–3]. In turn, physical inactivity creates a negative cycle marked by a further decline in physical function as the disease progresses. Importantly, physical activity and exercise positively affect motor capacity in PD [4, 5] with strong evidence that exercise improves gait, muscle strength, balance, and endurance [6, 7], even in the long-term [8].

Alongside physical decline, deterioration in cognition commonly affects cognitive domains including executive function [9], working memory, and memory [10]. Cognitive training is an approach that uses guided practice on structured cognitive tasks with the direct aim of improving or maintaining cognitive abilities. It can be designed to target either one or multiple domains simultaneously [11]. Moreover, cognitive training is efficacious and improves cognition in PD, with positive effects on the specific domains executive function, processing speed, and working memory [12]. In the context of computerised cognitive training, positive impact on global cognition has been observed, suggesting both the efficacy and feasibility of such training [13]. Cognitive training is also proposed as an intervention strategy with potential to alleviate motor symptoms, especially freezing of gait, in PwPD [14–16]. Planning and decision making in particular, are important tasks involving executive function, and often require the ability to perform two tasks simultaneously, known as dual tasking [17]. When performing motor-cognitive dual tasking, PwPD have been shown to shift their focus from the given motor task to the cognitive task, increasing their risk of falling [18]. However, dual-task gait capacity can be improved through exposure to motor-cognitive training [19], whereby most interventions have been performed in the clinical setting. It remains unclear whether motor-cognitive training is feasible for PwPD, when performed in the home environment.

Electronic health (eHealth) technology, enables health care to be provided outside standard clinical settings [20]. eHealth methods are feasible in PD [21, 22] and beneficial for older adults [23]. A recent emergence of home training interventions among PwPD, with varying levels of remote supervision, report positive effects [24, 25], strengthening the hypothesis that effective health care can be provided outside clinical settings, using eHealth. Importantly, eHealth is a future alternative to providing accessible long-term support for self-management of symptoms throughout disease progression [26, 27].

The maintenance of behavioural change regarding physical activity in the long-term requires a systematic application of behavioural change techniques [28, 29]. Physical activity levels in healthy populations can be positively impacted, with techniques such as goal setting and self-reporting of physical activity being frequently used [30, 31]. Additionally, eHealth provides a novel and promising means by which to communicate and implement behavioural change techniques to those in need of them [32].

The aim of this study was to assess the feasibility of an eHealth delivered home exercise intervention using motor-cognitive components among PwPD. Feasibility aspects investigated included (i) Recruitment capability, (ii) Acceptability and Suitability of the intervention and (iii) Demand and Safety of the intervention. Patterns of change in physical outcomes and levels of physical activity were also investigated. Results from this feasibility study will inform decision making prior to a future randomised controlled trial.

## Methods

This study has a feasibility design as it planned to investigate whether the method *can work* as a first step in evaluating a novel intervention [33]. The design also captures important feasibility parameters such as the process of data collection, capability of recruitment, acceptability, suitability of the intervention, as well as patterns of change in physical outcome measures [33, 34]. The study rational and design is guided by Medical Research Council (MRC) recommendations for developing and evaluating complex interventions [35], and also adheres to the Consolidated Standards of Reporting Trials (CONSORT, extension to pilot and feasibility trials) [33]. See Additional File 1 for the complete CONSORT checklist. Furthermore, specific feasibility outcomes were guided by proposed objectives for feasibility designs, as described by Orsmond and Cohn [36].

### Recruitment

People with PD were recruited at one primary care rehabilitation clinic in central Stockholm, Sweden, through social media, the Swedish Parkinson Association, and by contacting participants in earlier cohorts. No formal sample size calculation was performed, but approximately 12 participants are considered suitable for inclusion in stage 1 clinical rehabilitation feasibility trials [37]. Participants were eligible for inclusion if they [1] had a diagnosis of idiopathic PD confirmed and documented (as International Classifications of Diseases (ICD-10) code G20.9) by a neurologist [2], were ≥ 50 years of age [3], were assessed as Hoehn and Yahr Stages 1–3 [4] were able to ambulate indoors without a mobility aid, and [5] had a stable anti-Parkinson’s medication regime three months prior to inclusion. People were excluded if they had cognitive difficulties and scored ≥ 21 on the Montreal Cognitive Assessment (MoCA©), did not have access to Wi-Fi in the home, were unable to travel to the clinic for assessments or had significant or uncorrected impairment of hearing or vision. The study was performed in accordance with the Declaration of Helsinki and was approved by the Swedish Ethical Review Authority (2020–03655). The trial was registered on Clinicaltrials.gov, NCT05027620, 2021-08-30. All participants were given verbal and written information concerning the study and signed written informed consent prior to trial commencement. Authors of this study have received permission to use MoCA©.

### Intervention

The home training intervention targeted functional strength, cardiovascular fitness, flexibility, and motor-cognitive function. The eHealth intervention was delivered using the ExorLive® application, which was already in use in the clinic in question and is commonly used within Scandinavian rehabilitation systems to supply physiotherapists with a comprehensive exercise bank. Exercise programs can be created and provided either in paper format, which is most commonly used, or using the digital application, and consist of filmed exercises or programs.

The program was designed to be performed during 20-minute sessions, 3 times weekly, over 10 weeks using the ExorLive® app, viewed from a digital tablet. The exercises were individually adapted according to baseline function, and the level of challenge was increased progressively. The physical exercise programs were set to three different levels of difficulty: M1, M2 and M3, where M1 had the lowest level of motor challenge and M3 the highest. Progression of the motor challenge occurred halfway through the program, at week six.

**Fig. 1:**
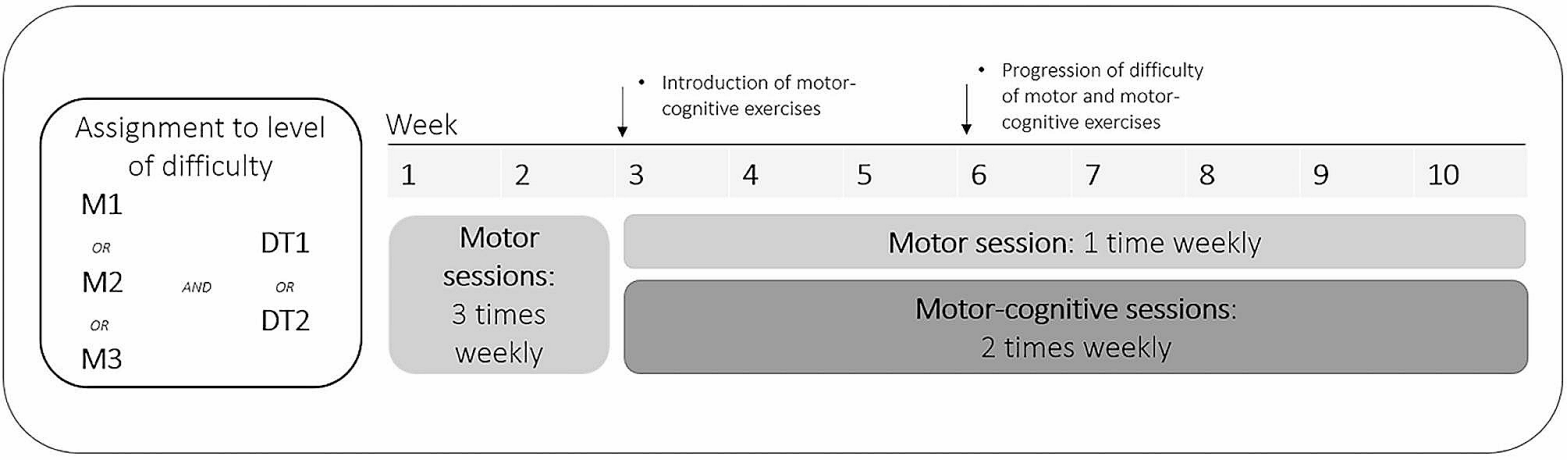
Intervention process, from program assignment to levels of difficulty. Levels M1-M3 represent the motor level, where M1 was the lower motor level and M3 the highest. Levels DT1 and DT2 represent the cognitive level, where DT2 was the higher level

Motor-cognitive components were introduced at week three, during two of the three weekly sessions and these exercises varied from 2 to 6 minutes in duration. Cognitive tasks were designed based on current evidence and targeted aspects of executive function, processing speed, and working memory [38, 39]. Within these three cognitive domains, exercises were designed in five subdomains involving tasks such as: reciting letters or words, verbal recalling, digit span, counting categories, and counting movements. The complexity and duration of the motor-cognitive components also progressed between weeks 3 − 6. The motor-cognitive exercises were set at two levels of difficulty: Dual-task level 1 (DT1) and dual-task level 2 (DT2), where DT2 had a higher level of cognitive challenge. For the cognitive domains verbal recalling and digit span, DT1 challenge progressed to a maximum of four words and three digits, respectively. At the higher DT2 level, these domains progressed to a maximum of five words and digits. Participants were streamed into the different motor-cognitive levels based on baseline cognitive score (MoCA©) and dual-task performance, during a 2-minute walk test with dual task-condition and the Timed Up and Go cognitive test. The intervention process is illustrated in Fig. 1. A specification of motor-cognitive levels and progression is presented in Additional file 2.

Additionally, with the aim to increase physical activity in everyday life during the 10-week period, behavioural change techniques were applied by setting personal goals with each participant at program outset. This process was performed using individual telephone interviews with all participants, and goals were revisited at 5 weeks and evaluated at the end of the intervention period.

Weekly follow-ups with a physiotherapist, using phone calls or messages in the app, assessed whether the level of both motor and motor-cognitive sessions were appropriate for the respective participant. Participants were encouraged to communicate potential difficulties they experienced using the app.

### Data collection relating to feasibility outcomes

The feasibility outcome *Recruitment capability*, was assessed in terms of how well the recruitment targeted the intended population, in relation to participants’ physical and cognitive measurements.

Following each exercise session, participants completed a home exercise diary (HED). Questions from the HED are presented in Table 1 and were constructed to capture data concerning the feasibility outcomes *Acceptability and Suitability*. Participants rated their level of perceived exertion after each session, using the Borg RPE scale, 6–20 [40]. A weekly question concerning falls captured the feasibility outcome *Safety*.

*Demand* was assessed using HED data concerning participants’ reported adherence and further validated with adherence data from sessions marked as “complete” in the application.

**Table 1:** Overview of questions in the home exercise diary in relation to feasibility outcomes

Primary feasibility outcome	Question	Possible answers
Acceptability	Did you enjoy the exercise session?	Yes; Neither; No
Suitability	What was your level of exertion?	Rating scale: Borg RPE, 6–20, where 6 = no exertion at all, and 20 = maximal exertion
Acceptability/Suitability	How did you perform during the motor-cognitive session?	Very well; Well; Neither; Badly; Very badly
Suitability	Were there any difficulties during the motor-cognitive session?	No; The instructions were unclear; I could not see the instructions; It was too quick; I forgot to move; Others
Suitability	How challenging was the motor-cognitive session for you?	Rating scale from 0–10, where: 0 = not challenging at all, and 10 = extremely challenging
Safety	Have you fallen? If so, how, and when did you fall?	Yes; No

### Outcome measurements relating to motor and cognitive function

Demographic data regarding age, gender, height, weight, years of education, disease duration, PD medication intake, and self-reported falls during the past six months were collected at baseline measurements. To evaluate global cognition the MoCA© [41] was performed at baseline. Executive function was assessed using both phonemic and semantic Verbal Fluency [42] and the Trail Making Test (TMT), part 2 and 4 [42]. The TMT also captures attention and working memory [42]. To evaluate global lower limb strength, the 30 second chair stand test [43] was performed. Balance and motor function were assessed at baseline and post-intervention, using the Mini Balance Evaluation Systems Test (Mini-BESTest) [44, 45], 10-meter walk test [46] and the 2-minute walk test (2MWT) [47] performed in single and dual-task conditions. During dual-task conditions, an auditory Stroop task [48] was performed with participants hearing the words “high” or “low” at high or low frequency and responding whether the frequency was high or low. The auditory stimuli were set to occur with a 1.5 to 2 seconds interval with an incongruity set at 50%. The auditory Stroop task was also performed as a single task while sitting. To capture temporo-spatial gait characteristics during walking, six wearable sensors (APDM, Inc) [49] were worn during the 2MWT (single and dual-task), Timed Up and Go and Timed Up and Go cognitive. The sensors were placed on both lower and upper body: both feet and wrists, chest, and posterior trunk, using elastic bands. Wirelessly, data was streamed to a laptop using the Mobility Lab Software (APDM, Inc).

Physical activity was captured with a waist-worn accelerometer ActiGraph GT3X (ActiGraph, Pensacola, FL, US). Frequency was recorded on three axes of 30 Hz. Epochs of 60 seconds were used for determination of steps per day and time spent in different levels of physical activity, using validated cut-point for older adults, where sedentary behaviour were set to < 100 counts per minutes, light-intensity physical activity (LIPA) between 100 and 1040 counts per minute, and moderate to vigorous intensity physical activity (MVPA) with ≥ 1041 counts per minute [50]. A minimum of four valid days up to seven days with 10 hours of wear-time a day was required for inclusion in the analysis. If there was 60 minutes without counts, data was removed as it was considered non wear time [50].

Patient-reported outcome measures were collected via questionnaires for the following domains and outcomes: balance confidence, the Activities-specific Balance Confidence Scale [51]; walking ability, Walk-12G [52]; self-efficacy for exercise, the Exercise Self-Efficacy Scale, Swedish version [53]; health related quality of life, EuroQol 5 Dimensions, three levels [54] and disease-specific health and quality of life, the Parkinson’s Disease Questionnaire (PDQ-39) [55]. To evaluate the demand and usability of the eHealth tool, the 10-item System Usability Scale (SUS), was used as it is widely used and previously validated [56]. In the SUS, a score above 70 is considered as good and a score above 90 represents excellent usability [57].

### Statistical analysis

The software IBM SPSS Statistics 28 for Windows was used for statistical analysis. Descriptive statistical analysis was performed to assess normality distribution, looking at percentages and averages of feasibility outcomes. Normality was assessed with skewness values and visual inspection of QQ-plots and histograms. For normally distributed data, mean and standard deviation (SD) was used. For non-normally distributed data, instead median and the interquartile range (IQR) was presented.

## Results

### Recruitment capability

Data collection occurred between September 2021 and January 2022. A total of 33 PwPD were screened, fifteen of whom met the inclusion criteria, were assessed at baseline and commenced the trial (mean age 68.5 years, 47% women). All participants received active levodopa treatment. One participant did not report details of the levodopa dosage and was excluded from the calculation of levodopa equivalent daily dose (LEDD). Increased LEDD was reported by four participants post-intervention.

**Fig. 2:**
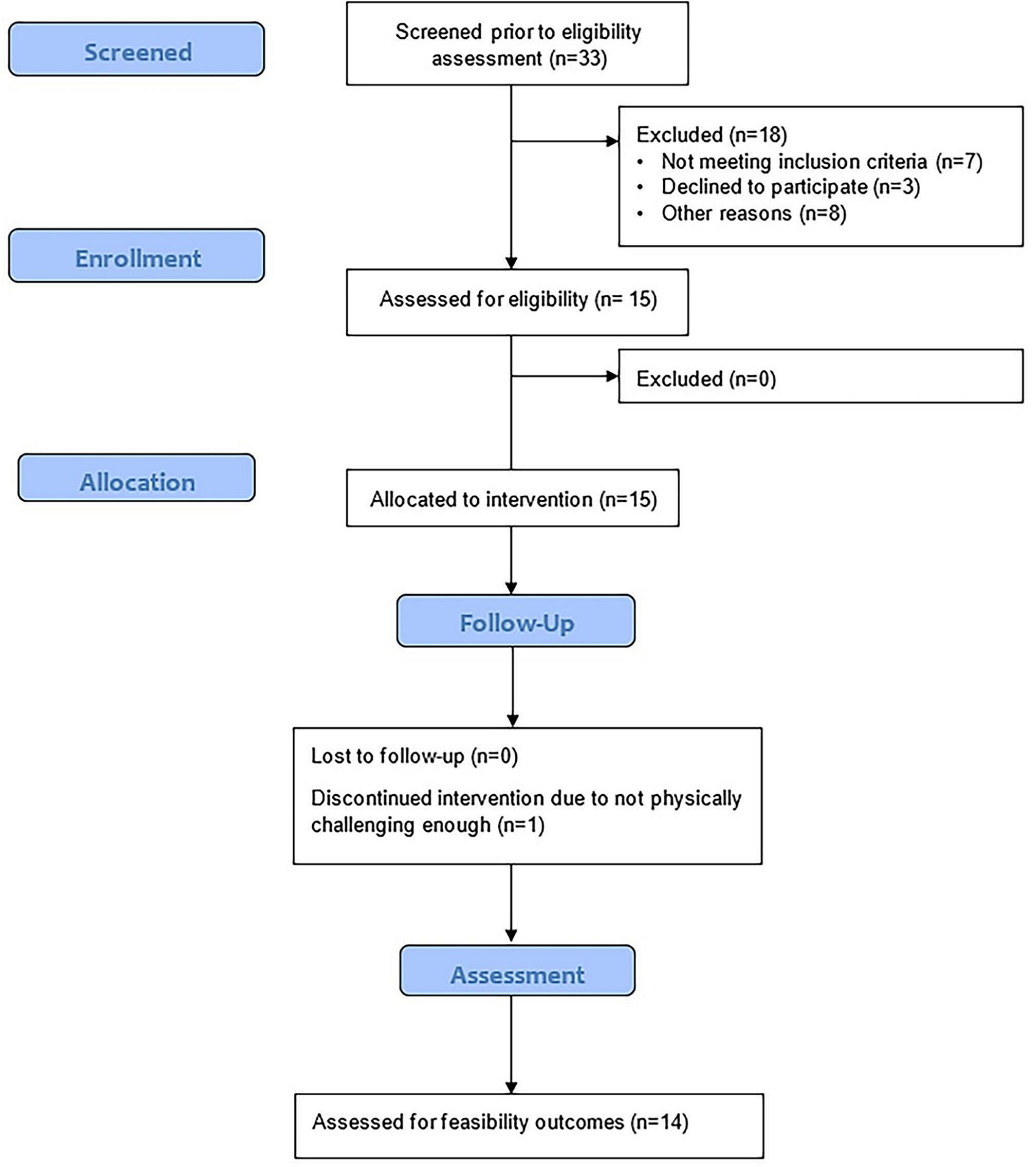
CONSORT 2010 statement extension to randomised pilot and feasibility trials [33]

One participant dropped out during the intervention period, declaring the intervention as not physically challenging enough. See Fig. 2 for a flow diagram. Participants demographic characteristics are summarised in Table 2.

**Table 2:** Descriptive characteristics of study participants (*n* = 15)

Characteristics, mean (SD), unless otherwise stated	
Sex, female, n (%)	7 (46.7)
Age, years	68.5 (9.0)
Body Mass Index, kg/m^2^	24.1 (3.3)
Hoehn and Yahr, 0–5	2.5
2, n	7
3, n	8
Years with diagnosis, median (IQR)	7.1 [5]
LEDD, mg/day, median (IQR)	555 (608)
Self-reported falls, last 6 months	1 (2.3)
Years of education	15.1 (3.0)
Living conditions, n (%)	
Cohabitating	10 (66.6)
Living alone	5 (33.3)
House	5 (33.3)
Apartment	10 (66.6)
Familiar with digital technology, n (%)	
Yes	10 (66.6)
Partly	3 [20]
No	2 (13.3)

*Abbreviations* LEDD: Levodopa equivalent daily dose; IQR: Interquartile range

### Acceptability and suitability of the eHealth intervention

In relation to *Acceptability* of the intervention, 64% of the motor sessions were rated as “enjoyable” and 29% were rated as “neither”. The motor-cognitive sessions were rated as “enjoyable” in 52% of cases, while 26% of the sessions were rated as “neither”. Additionally, 71% of study participants rated the system as 70 or higher on SUS, which indicated good perceived usability. In total, half of participants rated the system 82.5 or higher, indicating higher usability.

Regarding adherence, 86% of all (420) exercise sessions were reported as completed by participants in the HED. In comparison, 85% of the exercise sessions were marked as completed in the training app.

In relation to *Suitability*, participants rated their average level of exertion as light (11–12) on the Borg RPE scale. However, higher average ratings (12.9) were seen towards the end of 10-week period, in comparison to week 3 (11.0). The total number of adaptations made by the research team in participants exercise programs concerning the motor levels varied from one to five adaptations during the intervention period, with an average of three changes.

Regarding suitability of the cognitive components, participants reported an average rating of three out of ten for the level of challenge of the exercises. Participants in group DT1 (lower cognitive challenge) reported motor-cognitive tasks as challenging to a higher extent compared to DT2 (higher cognitive challenge), 5.2 in comparison to 1.9. No additional adjustments were made to the challenge of cognitive exercises throughout the intervention period.

Thirteen participants set a goal related to their physical activity or exercise and 54% of these were reported as fulfilled by the end of the intervention. One participant was already physically active to a high extent and one participant was not interested in formulating a goal regarding physical activity.

Most participants (75%) did not report difficulties during the exercise sessions. The most frequently reported issue was lack of time between the exercises (reported 17 times). Other reasons reported were forgetting to perform the motor task (reported 11 times), the instructions were not visibly clear enough (reported 10 times) and that the instructions were unclear (reported 6 times).

The majority of participants reported performing the motor-cognitive exercises “very well” (49.2%) or “well” (34.2%). Less than 8% of the sessions were reported as having been performed “badly” (5.8%) or “very badly” (1%), indicating that suitable cognitive tasks were assigned for the study sample.

### Demand and safety

No falls or other adverse events were reported in conjunction with the exercise sessions during the 10-week intervention period. However, a total of four falls were reported during activities of daily life by three study participants, during this time period. Another safety aspect reported by participants was that they did not always have enough time to move from one position to another during the program.

### Patterns of change in physical outcomes

**Fig. 3:**
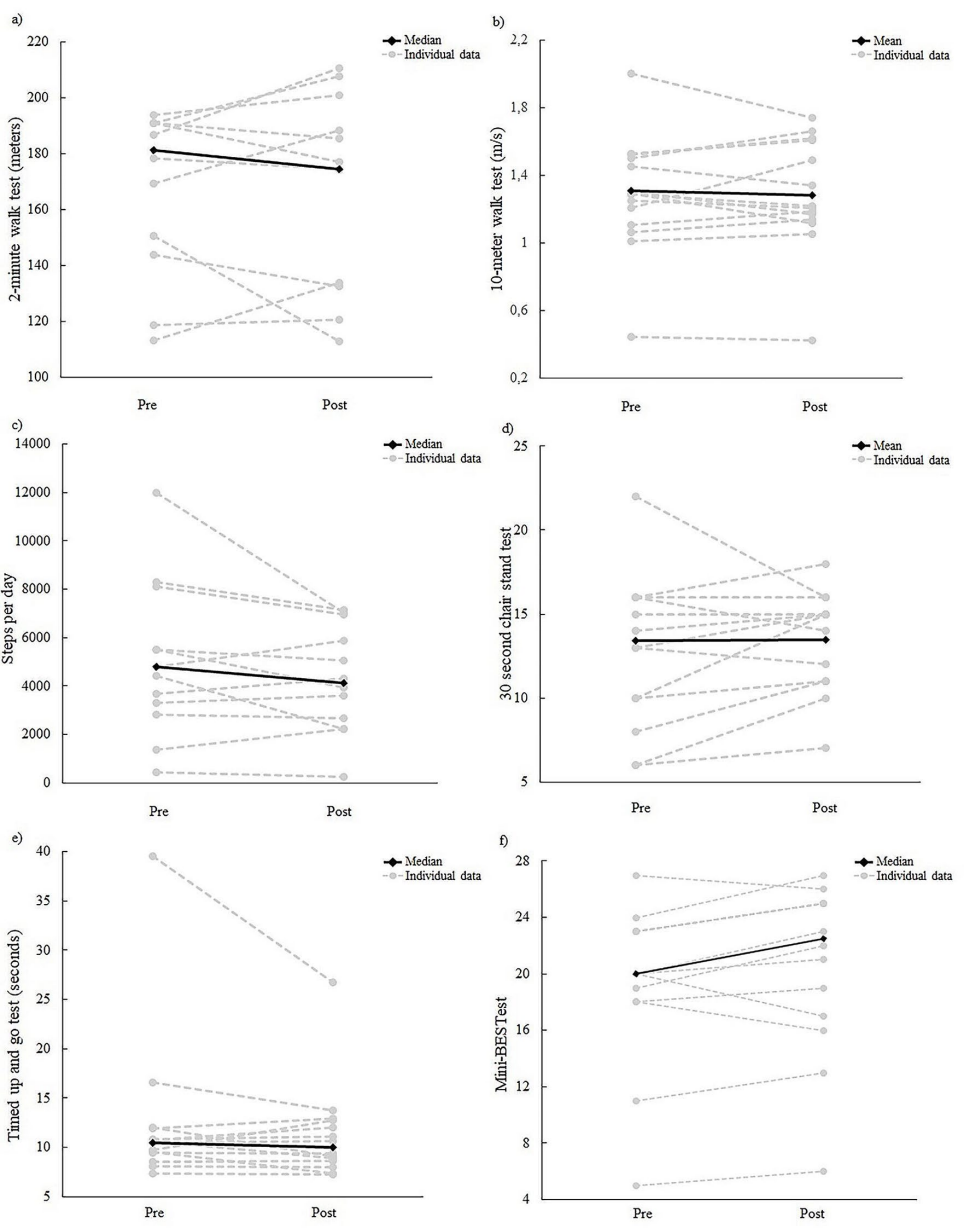
Patterns of change in (**a**) 2-minute walk test, (**b**) 10-meter walk test (usual gait), (**c**) steps per day, (**d**) 30 s chair stand test, (**e**) Timed up and go test, (**f**) Mini Balance Evaluation Systems Test

Both positive and negative patterns in physical outcomes were observed over the 10-week period, see Fig. 3. The absolute mean/median values at baseline assessment and post-intervention can be found in Additional file 3, Table 1.

Median physical activity levels were lower by the end of the intervention period in terms of daily steps and minutes spent in physical activity. Data from thirteen participants was collected at baseline and from twelve participants post-intervention, details presented in Additional file 3, Table 2.

Accuracy levels for Auditory Stroop were high both at baseline and post-intervention and demonstrated a ceiling-effect, see Additional file 3, Table 1 for details.

## Discussion

This feasibility study assessed the Recruitment capability, Acceptability, Suitability, Demand, and Safety of a novel motor-cognitive home training program using eHealth among PwPD. High adherence, acceptance and safety of the intervention were observed. In relation to intervention suitability, findings revealed that the level of physical challenge of the intervention was insufficient. The motor-cognitive levels were seen as suitable although an increase in duration would be beneficial in a future trial.

The recruitment strategy was successful as it resulted in the targeted number of participants, but the sample unfortunately had a large variation in relation to motor capacity and physical activity level. Recruitment aimed to reach PwPD, at mild to moderate disease stages who were physically inactive. Baseline measurements showed high overall levels of physical function while objectively measured physical activity level (steps per day) was low. The average physical activity level was below 5000 steps per day, in line with previous research among PwPD [1, 58, 59], who are more sedentary and take less steps than healthy age-matched controls [60]. Seasonality is previously reported to affect physical activity behaviour [61, 62], a factor which could be reflected in our findings, as baseline measurements were conducted in September and post measurements performed in the colder and darker winter months December/January. Additionally, participants reported “bad weather” as one reason why their physical activity goals were not reached by the end of the study period.

In terms of intervention acceptability, participants reported the majority of motor sessions as being “enjoyable”, reflecting satisfaction with the exercise program. However, compared to the motor sessions, fewer motor-cognitive sessions were rated as “enjoyable”, Interpretation of this finding is complex as lower enjoyment could reflect difficulties in dual-task performance. Additionally, cognitive tasks were more commonly reported as being difficult among those assigned the lower level of cognitive challenge (DT1). Self-reported adherence to the sessions was high when viewed in relation to other home-based trials for PwPD [24, 25, 63] and could further reflect participant satisfaction. This finding is positive considering the fully unsupervised nature of the program and that adherence to home exercise in PD is known to be challenging, especially in the long-term [24, 64]. In awareness of the tendency to overreport home-training in previous PD trials [65], we validated self-reported adherence data with ExorLive® data usage regarding the total number of completed sessions and observed comparable adherence. This high adherence could be a result of the regular and accessible contact with a physiotherapist during the 10-week period.

We used the SUS to evaluate how participants rated using the application. Most participant ratings indicated high usability [66], which strengthens continued use of the application in intervention delivery. The evaluation of System Usability enables future improvements regarding implementation of application solutions, which is important as using an application suitable to patient group, motor and cognitive levels is noted to be of relevance for successful end-user experience [67].

A major finding relating to intervention suitability, was that the level of challenge of home exercises was insufficient to improve walking capacity and physical activity levels. Existing evidence for dose-response effect of exercise in PwPD indicates the need for higher challenging strength and cardiovascular exercise [68, 69]. Feasibility findings highlighted areas for future improvement, such as increasing the duration and intensity of the video-sessions, as well as further refinement of the process where individual physical activity goals are set. Such measures should enable future participants to reach the recommendation of 150 min of moderate physical activity and exercise each week [70]. The Borg RPE scale is well-used for rating the level of exertion and correlates to heart rate [71, 72] and blood lactate concentration [72]. A study which aimed to validate the Borg RPE scale among PwPD, observed high correlation between heart rate and rated level of perceived exertion, however, higher ratings of the RPE scale were seen, indicating workload being experienced as more challenging in PwPD [73]. This study aimed for a level of exertion around level 13, translated to “somewhat hard” or 130 bpm [40]. Clearly, the dose was not sufficiently high for these participants as the average ratings on Borg RPE scale were lower than anticipated, which in turn required a series of adaptations and additions to be made to participants training programs during the 10-week period. It is also of note that the participant who discontinued the intervention did so because the exercise sessions were not physically challenging enough. Considering the feasibility outcome Suitability, the future intervention should be adapted to challenge physical capacity to a greater extent, in order to improve walking capacity. This focus should be reflected in the choice of the main outcome.

As the main goal of a feasibility study is to evaluate if an intervention and its processes can work, inferential statistics are not always performed [74]. Although this study was not powered to detect statistical differences in measured outcomes, both positive and negative patterns were observed in physical outcomes. These results should be interpreted with caution as the sample size is small. In relation to suitability of the cognitive components of the intervention, the cognitive tasks were generally reported as “not challenging”. Furthermore, that participants reported having insufficient time to perform tasks, signals the need for further adjustments to be made to better account for PD-specific motor impairment.

A ceiling effect was observed in the auditory Stroop test during baseline and post-intervention assessments. This finding highlights the need to increase the complexity of the dual-task test in order to better capture participants’ actual dual-task capacity, prior to a large-scale trial. This could be accomplished by increasing the ratio of incongruent stimuli.

Fulfillment of the individual physical activity goals was low, with half of participants reaching their set goals. However, the goals had a wide variation, ranging from improvements in functional strength to physical activity. In a future trial, goal setting should be narrowed down and more distinctly guided toward achieving specific physical activity levels. Levack et al. observed the complexity of goal setting in rehabilitation and how current evidence is inconclusive whether goal setting improves physical abilities or levels of physical activity [75]. Although, setting short-term goals in an inpatient clinical setting has earlier been found to be of clinical relevance among a neurological population [76].

The very low rate of dropouts possibly reflects the suitability and acceptability of the intervention but could also reflect the high capacity of the sample. In a systematic review, among older adults, people with greater physical or cognitive difficulties were found to dropout from studies to higher extent [77]. No adverse events occurred during the intervention, indicating the safety of sessions and possible advantages of the individual tailoring of the motor and cognitive challenge in accordance with baseline capacity. Previous trials also report home exercise interventions as safe for PD, both digital and non-digital [25, 78], although those with freezing of gait and those with cognitive decline might require greater supervision [25].

## Strengths and limitations

To our knowledge, this is the first study in PD that tests the feasibility of a motor-cognitive exercise intervention in the home using eHealth. A major strength of this study was the individual tailoring of exercises to motor and cognitive function, a method strongly supported in literature [79]. Additionally, continuous changes to the intervention were enabled during the intervention period, highlighting the strength of adopting a feasibility design in the early stage of intervention development [35]. Another strength regarding adherence, is the validation of self-reported adherence using retrieved data from the application. However, neither source of adherence data can fully ensure that participants trained as intended nor the quality of their performance. There were also limitations as this study did not test the feasibility of the randomisation process or the control group condition – necessary design features in a future efficacy trial. Although suitable in feasibility studies, the small sample size limits the ability to fully evaluate the recruitment process.

## Conclusion

This eHealth home exercise intervention for PwPD targeting motor-cognitive function was safe and feasible in terms of Recruitment capability, Acceptability, Safety and Demand. Suitability findings showed the need to increase the intensity of physical challenge of the intervention before commencing with a large-scale efficacy trial.

### Electronic supplementary material

Below is the link to the electronic supplementary material.

Additional File 1: CONSORT checklist.

Additional File 2: Specification of the motor-cognitive levels and progression.

Additional File 3: Physical, neuropsychological and self-reported outcomes.

## Data Availability

The datasets generated and/or analysed during the current study are not publicly available due to Swedish legislation but are available from the corresponding author on reasonable request.

## Abbreviations

DT1: Dual-task level 1
DT2: Dual-task level 2
eHealth: Electronic health
HED: Home exercise diary
LEDD: Levodopa equivalent daily dose
LIPA: Light-intensity physical activity
Mini-BESTest: Mini Balance Evaluation Systems Test
MoCA: Montreal Cognitive Assessment
MVPA: Moderate to vigorous intensity physical activity
PD: Parkinson’s disease
PDQ-39: the Parkinson’s Disease Questionnaire
PwPD: People with Parkinson’s Disease
SUS: System Usability Scale
TMT: Trail Making Test
2MWT: 2-minute walk test
