# Supplementary material for: Feasibility of a novel eHealth intervention for Parkinson’s disease targeting motor-cognitive function in the home
Source: BMC Neurol. 2024 Apr 5;24:114. doi: 10.1186/s12883-024-03614-2 (PMC10996106; doi:10.1186/s12883-024-03614-2)
Supplement: Supplementary file 2 — Additional File 2: Specification of the motor-cognitive levels and progression. [file 12883_2024_3614_MOESM2_ESM.pdf]

Additional file 2.

Level D1	Week 1	Week 2	Week 3	Week 4	Week 5	Week 6	Week 7	Week 8	Week 9	Week 10
First session			Counting categories	Counting movements	Verbal recalling	Digit span forward<br>Reciting objects	Counting categories<br>Digit span forward	Reciting letters/words<br>Verbal recalling	Counting categories<br>Digit span forward	Reciting letters/words<br>Verbal recalling
Second session			Counting movements	Verbal recalling	Reciting letters/words	Digit span forward<br>Reciting objects	Counting categories<br>Digit span forward	Reciting letters/words<br>Verbal recalling	Digit span backward<br>Counting categories	Verbal recalling<br>Reciting letters/words
Total time spent in dual task condition			5 min, 35 s	6 min, 19 s	9 min	12 min	9 min	12 min	12 min	11 min

Counting categories: Starting with counting only one category up to two combined categories, i.e., shape and colour of object.

Counting movements: counting every or every other movement being performed within a set time frame.

Verbal recalling: Starting with three words to remember up to four words.

Reciting letters/objects: Reciting letter, words or objects.

Digit span: Starting with 3 ascending numbers to both ascending and descending numbers, some mixed, and up to 4 numbers to remember.

**Level D2**	Week 1	Week 2	Week 3	Week 4	Week 5	Week 6	Week 7	Week 8	Week 9	Week 10
First session			Counting categories	Counting movements	Verbal recalling	Digit span forward	Counting categories	Reciting letters/words	Counting categories	Reciting letters/words
						Reciting objects	Digit span forward	Verbal recalling	Digit span forward	Verbal recalling
Second session			Counting movements	Verbal recalling	Reciting letters/words	Digit span forward	Counting categories	Digit span backward	Verbal recalling	Counting categories
						Reciting objects	Digit span forward	Counting categories	Reciting letters/words	Digit span backward
Total time spent in dual task condition			5 min, 52 s	6 min, 19 s	9 min	12 min	9 min	12 min	12 min	12 min

Counting categories: Starting with counting only one category up to two combined categories, i.e., shape and colour of object.

Counting movements: counting every or every other movement being performed within a set time frame.

Verbal recalling: Starting with three words to remember up to five words.

Reciting letters, words or objects shown on screen.

Digit span: Starting with 3 ascending numbers to both ascending and descending numbers, mixed, and up to 5 numbers to remember.

Motor level: M1. All exercises instructed to be performed while standing with support from a chair.

Example of included exercises: Walking on the spot, boxing, bow and arrow movements, lunges, side steps, running on the spot, hip abduction, hip extension, sit-to-stand, heel raises, tandem standing, push-ups against wall, flexibility movements sitting on chair for both upper and lower body, weight shifting sideways.

Motor level: M2. Exercises instructed to be performed both while standing and laying on the floor. Some exercises instructed support from chair.

Example of included exercises: Walking on the spot, boxing, bow and arrow movements, lunges, side steps, running on the spot, hip abduction, clamshells, hip extension, sit-to-stand, heel raises, push-ups against wall, flexibility movements sitting on chair for both upper and lower body, weight shifting sideways.

Motor level: M3. Exercises instructed to be performed both while standing and laying on the floor. Some exercises instructed support from chair.

Example of included exercises: Running on the spot, boxing, bow and arrow movements, lunges, side steps, lunges sideways, hip abduction, clamshells, hip extension, squats, glute bridges, reversed sit-up, heel raises, push-ups on knees, flexibility movements sitting on chair for both upper and lower body, weight shifting sideways.

Increase in difficulty of motor level

For all three motor levels, time and repetition of exercises increased at week six.
