# Supplementary material for: Feasibility of a novel eHealth intervention for Parkinson’s disease targeting motor-cognitive function in the home
Source: BMC Neurol. 2024 Apr 5;24:114. doi: 10.1186/s12883-024-03614-2 (PMC10996106; doi:10.1186/s12883-024-03614-2)
Supplement: Supplementary file 3 — Additional File 3: Physical, neuropsychological and self-reported outcomes. [file 12883_2024_3614_MOESM3_ESM.pdf]

Additional file 3.

Table 1. Physical assessments, self-reported measures and neuropsychological assessments.

**Outcomes**, median (IQR) unless otherwise stated	Baseline (n=15)	Post-intervention (n=14)
**Physical assessments**		
2MWT single task, meters	181.2 (43.5)	174.6 (68.1)
2MWT dual-task, meters	165.6 (37.5)	166.5 (46.7)
10 MWT usual gait, m/s, mean (SD)	1.31 (0.4)	1.28 (0.3)
10 MWT fast gait, m/s, mean (SD)	1.82 (0.5)	1.86 (0.5)
Timed Up and Go	10.5 (3.4)	10.0 (2.3)
Timed Up and Go cognitive	12.4 (3.2)	12.9 (9.4)
30 Second Chair Stand Test, mean (SD)	13.4 (4.6)	13.5 (3.0)
Mini-BESTest	20.0 (5)	22.5 (8.3)
**Self-reported measures**		
PDQ-39, summary index	20.2 (22.1)	16.3 (22.4)
ABC, %	86.3 (26.3)	86.3 (29.6)
Walk 12	10.0 (13.0)	7.5 (9.8)
EQ-5D-3L		
Index	0.7 (0.2)	1.0 (0.4)
Visual Analogue Scale	62 (30.0)	70.0 (28.9)
ESES-S	29.0 (15.0)	22.0 (20.5)
**Neuropsychological assessments**		
Montreal Cognitive Assessment	26.0 (4.0)	-
Verbal Fluency*, raw scores		
Letter Fluency	38.0 (23.0)	41.5 (26.5)
Categorical Fluency	40.0 (16.0)	38.0 (18.8)
Categorical Switching	10.0 (5.0)	12.0 (4.3)
Trail Making Test*, seconds		
Part 2	42.7 (33.3)	39.9 (28.3)
Part 4	104.4 (101.5)	102.2 (101.4)
**Auditory Stroop, accuracy levels**		
Single task, %	100.0 (2.4)	100.0 (4.7)
Dual-task, %	98.8 (10.5)	100.0 (4.7)

Abbreviations: 2MWT: 2-minute walk test; 10MWT: 10-meter walk test; Mini-BESTest: Mini Balance Evaluation Systems Test (score between 0-28); PDQ-39: Parkinson's disease Questionnaire-39; ABC: Activities-specific Balance Confidence Scale (score between 0-100); EQ-5D-3L: EuroQol 5 Dimensions, three levels; ESES-S: Exercise Self-Efficacy Scale, Swedish version (score between 10-40).

*From Delis-Kaplan executive function system.

Table 2. Steps and levels of intensity.

**Outcomes**, median (IQR) unless otherwise stated	Baseline (n=13)	Post-intervention (n=12)
Wear time, minutes/day, mean (SD)	840.3 (101.3)	821.1 (95.6)
Steps per day	4798.4 (5145.8)	4125.8 (4343.5)
SB, minutes/day	626.0 (130.0)	632.8 (114.6)
LIPA, minutes/day	185.0 (127.1)	148.1 (107.5)
MVPA, minutes/day	27.4 (57.9)	28.1 (37.7)

Abbreviations: SB=Sedentary behaviour, LIPA=Light-intensity physical activity, MVPA=Moderate-vigorous-intensity physical activity
